# Unemployment during life can lead to metabolic syndrome in adult age. A 40-year follow-up of the Northern Swedish Cohort

**DOI:** 10.1093/eurpub/ckaf166

**Published:** 2025-09-17

**Authors:** Anne Hammarström, Maria Albin, Lars Alfredsson, Sofia Hernnäs, Katarina Kjellberg, Pekka Virtanen

**Affiliations:** Institute of Environmental Medicine, Karolinska Institutet, Stockholm, Sweden; Department of Epidemiology and Global Health, Umeå University, Umeå, Sweden; Institute of Environmental Medicine, Karolinska Institutet, Stockholm, Sweden; Institute of Environmental Medicine, Karolinska Institutet, Stockholm, Sweden; Department of Economics, Stockholm School of Economics, Stockholm, Sweden; Department of Finance and Economics, Hanken School of Economics, Helsinki, Finland; Institute of Environmental Medicine, Karolinska Institutet, Stockholm, Sweden; Centre of Occupational and Environmental Medicine, Region Stockholm, Stockholm, Sweden; Institute of Environmental Medicine, Karolinska Institutet, Stockholm, Sweden; Faculty of Social Science, Tampere University, Tampere, Finland

**Keywords:** Unemployment, Metabolic syndrome, Epidemiological research design, Life-course epidemiology, Gender

## Abstract

Little is known about the physiological outcomes of unemployment during life. The aim of this study is to analyse if exposure to unemployment during different age periods can lead to metabolic syndrome (MetS) in middle-aged men and women. Can sensitive periods be identified? Data from the Northern Swedish Cohort was used, a longitudinal study of school leavers from 1981. Over the 40-year period, the retention rate was 90%. MetS at age 56 was measured with clinical examinations, while the exposure was measured with retrospective matrices between follow-ups. Exposure was cut into tertiles in each age group, the contribution to risk from each month of exposure was also analysed, using logistic regression. Short-term exposure to unemployment in early teens (<12 weeks) as well as long-term exposure to unemployment during life (>24 months) was related to MetS among women. In addition, exposure to unemployment >24 months during age 22–30 was related to MetS in adult life among both men and women. A significant dose–response was found among men and women in the ages 22–30 and among women in the whole age period. All results were controlled for socioeconomic status, obesity and drinking, used as time-dependent confounders. Our study showed that long-term exposure to unemployment during life can lead to MetS in adult age among women. Sensitive periods were identified in young age among both men and women. Our findings can be understood as a maladaptive response to chronic stress over life becoming embodied as MetS in adult life and calls for offensive, age-adjusted gender-sensitive interventions on the labour market.

## Introduction

The rapid increase in metabolic syndrome (MetS) is emerging as a significant public health concern worldwide [1]. MetS is a clustering of metabolic disorders including dysglycaemia, central adiposity, hypertension, and dyslipidaemia. MetS is strongly predictive of cardiovascular (CV) disease [2], independent of age among men while decreasing with age among women. Thus, both gender and age should be considered when analysing CV risks.

MetS develops during life and therefore, there is a need for a life-course perspective in analysing its determinants [3]. Sensitive periods are defined as time windows when an exposure has stronger effects on the outcome compared to other times [3]. The other main life-course model of importance in this article is accumulation of risk.

The traditional biomedical model has embraced the notion that individual behaviour (such as alcohol consumption) induces CV risks, but ignored the contextual impact of, e.g. working life [4]. Unemployment reflects the inability of the labour market to generate employment for people who are actively seeking for a job. Unemployment represents a major public health problem for men and women with mental health consequences [5, 6], increased mortality risk [7] as well as deteriorated health behaviour (alcohol and smoking, analysed for men and women together) [8]. However, knowledge about physiological outcomes is largely lacking. We found only one meta-analyses in PubMed in the search for all meta-analyses and systematic reviews about unemployment (performed in August 2025). The meta-analyses found significant results for exposure to unemployment and both prediabetes and type 2 diabetes identical in men and women [9]. However, 11 out of the 12 studies measured unemployment cross-sectionally and the measures of both exposure and outcome were heterogenous. Thus, the knowledge gap is still large.

From a public health perspective, identifying risk factors, such as MetS [10], enables preventive measures of CV diseases before permanent damage has occurred. Moving focus from the individual to the environment will improve possibilities for primary prevention.

Few original articles have been published after the meta-analysis. One about unemployment and metabolic outcomes shows that disadvantaged employment trajectories (especially unemployment exposures) up to age 45 are related to increased allostatic load among men and women [11]. The study, based on the French cohort CONSTANCES, makes it probable that there are relations even though causality cannot be claimed. The study could not control for reverse causation or for confounders related to parental conditions during upbringing. In addition, the results are likely to be underestimated as groups with high degree of weak labour market attachment were not included.

A study from the same French cohort suggests that exposure to unemployment has an adverse effect of its own on metabolic risk factors and CV events, when taking gender, social position, and work environment into account [12]. The study has several limitations including self-reported outcomes. Most importantly, the participation rate was extremely low (7.3%), leading to a non-representative selection of socially privileged cohort members.

Thus, the possible impact of unemployment on physiological outcomes like MetS appears to be largely missing. There is a need for longitudinal studies about unemployment during different periods of the life course and metabolic disorders, including information on early conditions and clinically measured as biomarkers as outcome. Therefore, the aim of this study is to analyse if exposure to unemployment during different age periods can lead to clinically measured MetS in middle-aged men and women in a 40-year follow-up of school leavers. Can sensitive periods be identified?

## Methods

### Study design

The study design is a prospective 40-year longitudinal school-leaver study from a municipality in Northern Sweden.

### Setting

The number of women in the workforce in Sweden is internationally high. During the study period it increased from 67% in the early 1980s to 85% in the 2020s.

The level of unemployment in Sweden during the study period varied from very low (3%–4%) during the 1980s, to up to 10% during the financial crisis in the 1990s. After the crisis, the rate of unemployment has never declined to earlier low levels but remained high. The proportion of employees with temporary contracts has fluctuated around 13% (1990 and 2024) [13].

### Participants

The Northern Swedish Cohort (NoSCo) includes all pupils who in 1981 attended the last year of compulsory school in a municipality in Northern Sweden (*n = *1083) [14]. The cohort has been followed with extensive questionnaires from 1981 (age 16), 1983 (age 18), 1986 (age 21), 1995 (age 30), 2007(age 42) and 2020 (age 55). The questionnaires collected detailed information about participants’ labour market status since the previous follow-ups.

All participants were invited to clinical investigations during the latest follow-up (in 2020, age 55). Due to the COVID-19 pandemic most of the investigations took place in 2021, at age 56. The investigations were performed by well-trained nurses according to the WHO MONICA manual [15] and included measurements of blood pressure of waist circumference, and of length and weight, as well as venous blood sampling after overnight fast. Well-established CV risk factors (triglycerides, total cholesterol, HDL cholesterol, and fasting glucose) were analysed from the samples. All analyses were performed at the same accredited lab at Umeå University Hospital.

### Sample

The participation rates have been exceptionally high throughout the 40-year follow-up. Of the original cohort from 1981 (*n = *1083), 1049 were still alive in 2020, of which 942 (89.8%) answered the questionnaire.

We have clinical data for 85.4% of the 942 participants and complete data on MetS for 78.7% (*n = *741) at age 56. *N = *741 was used in all analysis. An analysis has been performed of alcohol consumers at age 16 among NoSCo participants at age 42 (*n = *1008) compared to at age 56 sample. No significant differences were found (*P* = .79).

### Ethical approval

This study was performed in line with the principles of the Declaration of Helsinki. Ethical approval was granted by the Swedish Ethical Review Authority (Dnr 2020-01950 and Dnr 2024-00320-01). Written informed consent was obtained from all individual participants included in the study.

### *Outcome*: MetS at age 56

MetS was defined according to the International Diabetes Federation [16]: (i) central obesity, defined as waist circumference ⩾80 cm for women and ⩾94 cm for men, and (ii) at least two of the following: (i) low serum HDL-C (<1.29 mmol/l for women and <1.03 mmol/l for men), or specific lipid therapy; (ii) high serum triglycerides (⩾1.7 mmol/l), or specific lipid therapy; (iii) high blood pressure (⩾130 mm Hg systolic and/or ⩾85 mm Hg diastolic) or antihypertensive medication; and (iv) raised fasting glucose levels (⩾5.6 mmol/l) or diagnosed type 2 diabetes mellitus (T2DM).

### *Exposure* to unemployment

At the start of the study in 1981, a seminar about unemployment was held at all schools before the questionnaires were distributed. In addition, personal interviews were performed with all participants who reported having been unemployed between 1981 and 1986. During the seminar and the interviews, the PI ensured that unemployment was defined as being without work, being able to work, and actively seeking jobs. Thus, the same criteria was used as later was defined by ILO. The PI also got permission to receive register data about unemployment from the local employment office when needed. Also, the matrix described in the next paragraph helped participants to distinguish between unemployment on one hand and parental leave, sick leave, being out of the labour market etc on the other hand.

The questionnaires at age 18 and 21 asked for the number of weeks unemployed since the latest follow-up. The questionnaires at age 30, 42 and 55 years inquired about labour market status since the previous follow-up using a matrix format. This matrix displayed status options (representing the most relevant and significant statuses for the cohort’s life course and the historical context of the time) on the vertical axis and time (divided into half calendar years) on the horizontal axis [14]. Respondents who experienced more than one status during a half-year period were instructed to mark all applicable options. Unemployment exposure was calculated as six months if the respondent marked unemployment as their sole status. If two statuses were marked, exposure was assumed to last three months; if three statuses were marked, exposure was assumed to last two months. If data was missing, personal interviews were performed.

After summing-up the number of weeks or months during the periods, the exposure to unemployment was trichotomized into “ zero,” “short-term,” and “long-term” as follows: during the ages 16–17 the cut-off between short-term and long-term unemployment was set at 12 weeks, while for ages 18–21, 22–30, 31–42, and 43–55 the cut-off was set at a total of 24 weeks of unemployment. Additionally, cumulative exposure during the follow-up from age 16 to age 55 was categorized using 24 months’ cut-off.

A test–retest analysis of the matrix was conducted in 2008, in which phone interviews were carried out with a random sample of 100 participants regarding labour market position over the 12-year period 1996–2000. The test–retest analysis showed answers as in the questionnaires. In addition, there is increasing evidence that self-reported retrospective employment histories provide valid and reliable information [17].

### Confounders

Confounders, i.e. common causes of exposures and outcomes, were identified via directed acyclic graphs in which causal relations are identified. In longitudinal studies, time-varying confounders must be considered.

Low socioeconomic status increases the risk of both unemployment [18] and MetS [19]. It was measured at age 16 as parental working-class belonging. The occupations of both parents were classified according to the standard of that time as “working-class” or “non-working class.” At age 18–21, education was more appropriate to use. At age 18 low education was defined as not attending school. At age 21, low education was defined as lack of exam from upper secondary school. At age 30 and age 42, socioeconomic position was determined using the Swedish SEI classification, based on current occupation. Low social class was defined as unskilled, semi-skilled, and skilled workers.

Childhood obesity increases the CV risk in adult life [20] and is related to unemployment during life [21]. Data on height and weight of the participants were obtained from the school health records at age 15, from clinical investigation measurements at age 42, and from questionnaires at ages 18, 21, and 30. Body mass index (BMI) was calculated, and for ages 15 and 18, the cut-off for obesity was set at 95th BMI percentile [22], calculated separately for girls and boys, while a BMI > 30 was used as the cut-off for adulthood (ages 21, 30, and 42).

High alcohol consumption increases the risk of unemployment [23] as well as of MetS [12]. Alcohol intake was calculated based on questionnaire data on drinking frequency and quantity. At age 16, respondents were classified as either drinkers or non-drinkers. At ages 18, 21, 30, and 42, the criteria established by the National Institute on Alcohol Abuse and Alcoholism (NIAAA, link: https://www.niaaa.nih.gov/alcohol-health/overview-alcohol-consumption/moderate-binge-drinking) were used to categorise respondents as heavy drinkers (defined as >14 drinks per week for men and >7 drinks per week for women) or not heavy drinkers.

### Sensitive period

Life-course epidemiology defines a sensitive period as a limited time window when exposure has a stronger effect on later health outcomes than it would at other times [3]. By analysing the risk of MetS due to unemployment in different age-groups, while controlling for later unemployment [24], we identified possible sensitive periods.

### Statistics

Binary logistic regression analyses were used to examine the associations between unemployment, measured as three categories and uncategorized unemployment during six age-periods, and MetS at age 56. The analyses were adjusted for socioeconomic status, drinking, and obesity at the first year of the period and, to identify possible sensitive periods, for unemployment exposure after the period of interest. Men and women were analysed separately due to gender-sensitive outcomes and labour market position [2, 25, 26].

## Results

Descriptive data are provided in Tables 1 and 2. A high proportion of participants had MetS at age 56, with 40% of women, and 57% of men affected. The gender difference was due to higher prevalence in men of all components except central obesity. Exposure to unemployment during different age periods was quite equally distributed among men and women.

**Table 1.** Prevalence of metabolic syndrome and its components in NoSCo at age 56, analysed by gender

	Men (*n = *380)	Women (*n = *361)	*P* value^a^
Metabolic syndrome	57%	40%	<.001
Hypertonia	81%	65%	<.001
Waist obesity	78%	78%	.993
Hypertriglyceridemia	52%	26%	<.001
Low HDL cholesterol	29%	20%	.002
(Pre)diabetes	46%	33%	<.001

a Chi-square test for gender difference.

**Table 2.** Descriptive statistics of the study sample by age of the survey and gender

	Men (*n = *380)	Women (*n =* 361)
Age 16		
Working class father	52%	49%
Working class mother	56%	57%
Obesity	5%	5%
Drinking (>0 cl)	58%	62%
Age 18		
Not in education	11%	13%
Obesity	5%	5%
Heavy drinking	5%	1%
Exposure to unemployment in age 16–17		
0 weeks	91%	85%
1–12 weeks	4%	9%
>12 weeks	6%	6%
Age 21		
Low-level education	11%	15%
Obesity	2%	1%
Heavy drinking	9%	2%
Exposure to unemployment in age 18–21		
0 weeks	59%	56%
1–24 weeks	33%	37%
>24 weeks	9%	7%
Age 30		
Working class occupation	65%	53%
Obesity	5%	3%
Heavy drinking	8%	5%
Exposure to unemployment in age 22–30		
0 months	67%	66%
1–6 months	12%	16%
>6 months	21%	19%
Age 42		
Working class occupation	61%	53%
Obesity	16%	13%
Heavy drinking	9%	8%
Exposure to unemployment in age 31–42		
0 months	78%	75%
1–6 months	5%	8%
>6 months	17%	17%
Age 55		
Exposure to unemployment in age 43–55		
0 months	89%	88%
1–6 months	3%	2%
>6 months	9%	9%
Exposure to unemployment in age 16–55		
0 months	33%	29%
1–24 months	47%	54%
>24 months	20%	18%

Table 3 presents the relationships between exposures to unemployment during different age periods and MetS at age 56 among women. Long-term total exposure over the lifespan, as well as long-term exposure during the age period 22–30 were related to MetS in all models. Before adjusting for later unemployment, short-term exposure to unemployment at age 16–17 was related to MetS, although with wide confidence intervals. Dose–response analyses showed significant positive associations for the age groups 22–30, 31–42 (which turned significant after adjusting for later unemployment) and 16–55. The figures mean that the respective 4.4%, 1.9%, and 1.8% rise of the odds ratio per additional month of exposure is statistically significant. Overall, adjustments for later unemployment tended to lower the odds ratios but a sensitive period remained significant for the age group 22–30.

**Table 3.** The risk of metabolic syndrome among women aged 56 due to exposure to unemployment^a^ during life

Age	Exposure	%	OR(1)	OR(2)	OR(3)
16–17	Zero (*n = *307)	37	1	1	1
	Short-term (*n = *35)	57	2.29 (1.13–4.65)	2.12 (1.03–4.37)	1.85 (0.87–3.91)
	Long-term (*n = *19)	53	1.91 (0.75–4.83)	1.72 (0.66–4.46)	1.59 (0.60–4.26)
	0–40 weeks^b^			1.024 (0.986–1.064)	
18–21	Zero (*n = *200)	36	1	1	1
	Short-term (*n = *130)	43	1.38 (0.88–2.16)	1.35 (0.86–2.14)	1.30 (0.82–2.08)
	Long-term (*n = *25)	48	1.68 (0.73–3.87)	1.31 (0.54–3.20)	1.17 (0.47–2.89)
	0–72 weeks^b^			1.010 (0.991–1.029)	
22–30	Zero (*n = *237)	38	1	1	1
	Short-term (*n = *56)	32	0.79 (0.42–1.46)	0.72 (0.38–1.37)	0.71 (0.37–1.37)
	Long-term (*n = *68)	53	1.87 (1.09–3.22)	1.92 (1.10–3.36)	1.82 (1.02–3.25)
	0–54 months^c^			1.044 (1.015–1.073)	
31–42	Zero (*n = *271)	39	1	1	1
	Short-term (*n = *30)	33	0.79 (0.36–1.76)	0.93 (0.41–2.10)	0.95 (0.42–2.15)
	Long-term (*n = *60)	47	1.38 (0.79–2.43)	1.30 (0.72–2.33)	1.28 (0.71–2.29)
	0–108 months^c^			1.019 (1.001–1.037)	
43–55	Zero (*n = *318)	38	1	1	
	Short-term (*n = *8)	38	0.96 (0.23–4.11)	1.09 (0.25–4.67)	
	Long-term (*n = *34)	50	1.61 (0.79–3.27)	1.61 (0.86–3.31)	
	0–144 months^c^			1.004 (0.990–1.018)	
16–55	Zero (*n = *103)	35	1	1	
	Short-term (*n = *111)	30	0.79 (0.44–1.40)	0.73 (0.41–1.31)	
	Long-term (*n = *119)	50	1.89 (1.10–3.25)	1.84 (1.06–3.19)	
	0–179 months^c^			1.018 (1.008–1.029)	

a For the ages 16–17 the cut-off between short-term and long-term unemployment was set at 12 weeks. For the age groups 18–21, 22–30, 31–42 and 43–55 the cut-off was set to 24 weeks of unemployment. For the whole age period (16–55) the cut off was set to 24 months of unemployment.

b The estimate refers to each week of unemployment within the given interval.

c The estimate refers to each month of unemployment within the given interval.

OR(1): unadjusted odds ratio; OR(2): odds ratio adjusted for socioeconomic status, for obesity and for intake of alcohol at the baseline year of the age period; OR(3): odds ratio adjusted as OR(2) plus unemployment during later ages.

Among men (Table 4) long-term exposure to unemployment during the age period 22–30 was significantly associated with MetS in all models, while no significant results were found for exposure during other periods. The accumulated measure of total unemployment was also positive in this age group, and the figures means that 2.2% rise of the odds ratio per additional month of exposure is statistically significant. The results were boarder significant for dose response for total time of exposure.

**Table 4.** The risk of metabolic syndrome among men at age 56 due to exposure to unemployment^a^ during life

Age	Exposure	%	OR(1)	OR(2)	OR(3)
16–17	zero (*n = *346)	56	1	1	1
	short-term (*n = *14)	79	2.94 (0.81–10.7)	2.32 (0.62–8.69)	2.27 (0.60–8.60)
	long-term (*n = *20)	65	1.49 (0.58–3.83)	1.22 (0.46–3.21)	1.17 (0.42–3.25)
	0–92 weeks^b^			1.003 (0.980–1.027)	
18–21	Zero (*n = *221)	54	1	1	1
	Short-term (*n = *123)	62	1.39 (0.88–2.17)	1.38 (0.88–2.18)	1.31 (0.82–2.09)
	Long-term (*n = *34)	56	1.09 (0.53–2.25)	0.93 (0.40–2.19)	0.70 (0.28–1.77)
	0–116 weeks^b^			1.008 (0.991–1.024)	
22–30	Zero (*n = *253)	53	1	1	1
	Short-term (*n = *46)	59	1.26 (0.67–2.39)	1.26 (0.67–2.38)	1.29 (0.68–2.45)
	Long-term (*n = *81)	68	1.88 (1.11–3.18)	2.11 (1.21–3.67)	2.11 (1.19–3.75)
	0–72 months^c^			1.022 (1.001–1.044)	
31–42	Zero (*n = *298)	57	1	1	1
	Short-term (*n = *18)	50	0.76 (0.30–1.98)	0.65 (0.22–1.88)	0.59 (0.20–1.74)
	Long-term (*n = *64)	59	1.12 (0.64–1.93)	0.99 (0.55–1.75)	0.95 (0.52–1.71)
	0–114 months^c^			1.007 (0.992–1.022)	
43–55	Zero (*n = *335)	56	1	1	
	Short-term (*n = *10)	40	0.53 (0.15–1.90)	0.58 (0.16–2.10)	
	Long-term (*n = *32)	69	1.74 (0.80–3.79)	1.81 (0.80–4.12)	
	0–146 months^c^			1.001 (0.980–1.024)	
16–55	Zero (*n = *138)	51	1	1	
	Short-term (*n = *92)	58	1.32 (0.78–2.24)	1.24 (0.72–2.15)	
	Long-term (keep *n = *123)	61	1.51 (0.93–2.48)	1.35 (0.81–2.23)	
	0–285 months^c^			1.006 (0.998–1.014)	

a For the ages 16–17 the cut-off between short-term and long-term unemployment was set at 12 weeks. For the age groups 18–21, 22–30, 31–42 and 43–55 the cut-off was set to 24 weeks of unemployment. For the whole age period (16–55) the cut off was set to 24 months of unemployment.

b The estimate refers to each week of unemployment within the given interval.

c The estimate refers to each month of unemployment within the given interval.

OR(1): unadjusted odds ratio; OR(2): odds ratio adjusted for socioeconomic status, for obesity and for intake of alcohol at the baseline year of the age period; OR(3): odds ratio adjusted as OR(2) plus unemployment during later ages.

Similar analyses for the single components of MetS show significant results among women for adverse lipid profile (high triglycerides as well as low HDL cholesterol), see Supplementary Table S1.

## Discussion

### Key results

Our main finding is that long-term exposure to unemployment during life is related to MetS in adult life among women. Long-term exposure to unemployment during age 22–30 was related to Mets in adult life among both men and women. A significant dose–response was found among both genders in the ages 22–30 and among women in the whole age period. Boarder significant results were found among men.

### Limitations and strengths

The main limitation is the relatively small sample size. As a result, some of the confidence intervals are wide, which limit the precision of those findings. At the time when the cohort was initiated, the size was regarded as large and made it possible to reach the extraordinary response rate over 40 years, as well as the high participation rate in the clinical examinations. An indication of low bias between the two latest follow-ups was that early alcohol drinking did not differ between them.

The observations of an increased risk for MetS, especially among women, in the categorical analyses was supported by non-categorical analyses for age 16 to 55 in which the risk for MetS was estimated to increase with 1.8% among women for each month of unemployment, corresponding to an increase of 18% for 10 such months, which is not negligible. The corresponding risk estimate for men was lower (6%), and boarder significant.

The main strength of NoSCo is the longitudinal design, from before entering the labour market until midlife, which increases the possibility to control for reverse causality. The exceptionally high response rate was due to the energy from the principal investigator to reach everyone as well as thanks to the large interest for the study among the participants as well as from the local municipality. In addition, all who became unemployed directly after compulsory school were included into an interview study, with regular interview follow-ups during the project period. As compared to other studies, those with the highest risk of unemployment were therefore not lost during the follow-up [14]. Moreover, MetS was assessed by standardized clinical measurements.

### Interpretation

The findings among women are in accordance with the few existing studies in the field [11, 12]. Obesity is the driving force behind MetS, which in turn is mainly caused by an imbalance of energy intake (diet) and energy expenditure (physical activity) [1]. However, we controlled all models for obesity at the beginning of the age period. Overall, the findings remained stable after adjusting for the confounders.

With a life-course epidemiological design we analysed time-dependent accumulation of exposure (dose–response analyses) and sensitive periods. The longer the exposure, the larger the risk. A sensitive period for exposure to long-term unemployment was identified between age 22 and 30 among both men and women. This finding indicates that exposure during this age window has the strongest effect on the outcome during life. In earlier research we have identified youth unemployment between age 16 and 21 as a sensitive period for hypertension among 43-year-old women in NoSCo [27]. So, both studies identified sensitive periods for metabolic outcomes among women in relation to youth unemployment and calls for research about possible explanations to the gendered findings.

The results among teenage girls that short-, but not long-term exposure to unemployment is related to MetS in adult age are difficult to interpret. At this time, there were ambitious Active Labour Market Policy programs directed towards unemployed young people which could influence our findings as studies indicate the beneficial effects of these programs on mental health [28].

Our results point to the need to analyse possible mechanisms between unemployment during life and MetS. Unemployment is a stressful life event, due to both psychosocial and financial strain [29]. The acute stress reactions can become embodied as chronic stress despite re-employment. Chronic stress reverses the favourable effects of an acute elevation of cortisol levels and can lead to alteration of the physiological response to stress, resulting in negative metabolic processes during life such as MetS [30]. Our study adds to current knowledge of how unemployment during life may lead to processes of biological embodiment of chronic stress reactions.

The prevalence of MetS in our study (57% in men, 40% in women) was high but comparable to Nordic studies using the same definition. The prevalence of MetS at age 46 (measured around 2007) in the Northern Finnish birth cohort was 41% among men and 25% among women [31], which is higher than the prevalence of MetS in NoSCo, measured at age 42 in 2007 (33.6% among men, 19.1% among women). The prevalence was 23% (only reported for men and women together) in a similar age group and time in the HUNT study from Northern Norway [32]. It is well-documented that the prevalence of MetS increases with both age and time, so the high prevalence at age 56 in 2021 in our study seems generalizable to similar contexts.

We have used modern analytical causal inference frameworks [33], throughout the analyses including causal directed acyclic graphs to select our confounders. Our findings strongly suggest causality, but this is the first study of its kind so more research is needed before definite causality can be concluded.

### Generalizability

NoSCo has been shown to be largely representative of Sweden in relation to demographics and health status, except higher exposure to unemployment compared to other parts of the country during the 1980s [13]. Unemployment is common worldwide in the working age population. The few available studies in our field from France [11, 12] and in the only meta-analyses (from several continents) on diabetes [9] indicates the generalization of our findings. Compared to other parts of the world, Sweden had both a more generous welfare system and more active labour market policy—especially for young people—during the 1980s. If welfare and active labour market provisions mitigate the consequences of unemployment, our results that unemployment increased MetS might be viewed as a lower bound on the effects in contexts with less generous welfare systems.

### Policy implications

Our study contributes with scientific evidence on long-term metabolic consequences of unemployment during life. From a public health perspective, it is important to identify determinants of health which society can influence. Unemployment is such a determinant and is an indicator in several public health policies such as the European Core Health Indicators (ECHI) - European Commission (europa.eu). Despite several EU initiatives specifically addressing young people (e.g. The Reinforced Youth Guarantee and The European Pillar of Social Rights Action Plan), the rate of youth unemployment remains high in Europe. Our findings regarding sensitive periods in young men and women underpin the need for intensified/more efficient approaches. Using health as an indicator in all policy areas including the financial sector, as suggested by the Helsinki statement of “Health in all policies” could here be of special importance in primary prevention of unemployment and thus a healthier population.

## Conclusion

The study showed that long-term exposure to unemployment during life can lead to MetS in adult age among women. Sensitive periods were identified in young age among both men and women. Our findings can be understood as a maladaptive response to chronic stress over life becoming embodied as MetS in adult life and calls for offensive, age adjusted interventions on the labour market.

## Supplementary Material

ckaf166_Supplementary_Data

## Data Availability

Given restrictions from the ethical review board and considering that sensitive personal data are involved, the data that support the findings of this study is not possible to make freely available. Access to the data may be provided in line with Swedish law and after consultation with the Umeå University legal department. Requests for data should be sent to the corresponding author. Key points Long-term exposure to unemployment during life can lead to metabolic syndrome in adult age among women. Sensitive periods were identified in young age among both men and women. The findings can be understood as a maladaptive response to chronic stress over life becoming embodied as MetS in adult life and calls for offensive, age-adjusted gender-sensitive interventions among long-term unemployed.

## References

[1] Saklayen MG. The global epidemic of the metabolic syndrome. Curr Hypertens Rep 2018;20:12. 10.1007/s11906-018-0812-z 29480368 PMC5866840

[2] Vishram JK , Borglykke A, Andreasen AH et al MORGAM Project. Impact of age and gender on the prevalence and prognostic importance of the metabolic syndrome and its components in europeans. The MORGAM prospective cohort project. PLoS One 2014;9:e107294. 10.1371/journal.pone.0107294 25244618 PMC4171109

[3] Kuh D , Ben-Shlomo Y, Lynch J et al Life course epidemiology. J Epidemiol Community Health 2003;57:778–83. 10.1136/jech.57.10.778 14573579 PMC1732305

[4] Vasan RS , Benjamin EJ. The future of cardiovascular epidemiology. Circulation 2016;133:2626–33. 10.1161/CIRCULATIONAHA.116.023528 27324358 PMC4974092

[5] McKee-Ryan F , Song Z, Wanberg C et al Psychological and physical well-being during unemployment: a meta-analytic study. J Appl Psychol 2005;90:53–76. 10.1037/0021-9010.90.1.53 15641890

[6] Taylor P , Gringart E, Adams C. Psychological effects of unemployment across the lifespan: a synthesis of relevant literature. J Aging Soc Policy 2023;35:154–78. 10.1080/08959420.2022.2136918 36368775

[7] Roelfs DJ , Shor E, Davidson KW et al Losing life and livelihood: a systematic review and meta-analysis of unemployment and all-cause mortality. Soc Sci Med 2011;72:840–54. 10.1016/j.socscimed.2011.01.005 21330027 PMC3070776

[8] Amiri S. Smoking and alcohol use in unemployed populations: a systematic review and meta-analysis. J Addict Dis 2022;40:254–77. 10.1080/10550887.2021.1981124 34747337

[9] Varanka-Ruuska T , Rautio N, Lehtiniemi H et al The association of unemployment with glucose metabolism: a systematic review and meta-analysis. Int J Public Health 2018;63:435–46. 10.1007/s00038-017-1040-z 29170882

[10] Reinikainen J , Kuulasmaa K, Oskarsson V et al Regional and temporal differences in the associations between cardiovascular disease and its classic risk factors: an analysis of 49 cohorts from 11 european countries. Eur J Prev Cardiol 2024;31:569–77. 10.1093/eurjpc/zwad359 37976098

[11] Wahrendorf M , Chandola T, Goldberg M et al Adverse employment histories and allostatic load: associations over the working life. J Epidemiol Community Health 2022;76:374–81. 10.1136/jech-2021-217607 34625518 PMC8921582

[12] Sanchez Rico M , Plessz M, Airagnes G et al Cardiovascular burden and unemployment: a retrospective study in a large population-based French cohort. PLoS One 2023;18:e0288747. 10.1371/journal.pone.0288747 37459323 PMC10351739

[13] Ekonomifakta. Tidsbegränsat anställda. https://www.ekonomifakta.se/sakomraden/arbetsmarknad/sysselsattning/tidsbegransat-anstallda_1209217.html (15 September 2025, date last accessed) (in Swedish).

[14] Hammarström A , Janlert U. Cohort profile: the Northern Swedish cohort. Int J Epidemiol 2012;41:1545–52. 10.1093/ije/dyr118 21828110

[15] WHO*. MONICA Manual, Part I: Description and Organization of the Project.* https://www.thl.fi/publications/monica/manual/index.htm (15 September 2025, date last accessed).

[16] Alberti KG , Zimmet P, Shaw J. Metabolic syndrome—a new worldwide definition. A consensus statement from the international diabetes federation. Diabet Med 2006;23:469–80. 10.1111/j.1464-5491.2006.01858.x 16681555

[17] Wahrendorf M , Marr A, Antoni M et al Agreement of self-reported and administrative data on employment histories in a german cohort study: a sequence analysis. Eur J Popul 2018;35:329–46. 10.1007/s10680-018-9476-2 31105502 PMC6497685

[18] van Zon SKR , Reijneveld SA, Mendes de Leon CF et al The impact of low education and poor health on unemployment varies by work life stage. Int J Public Health 2017;62:997–1006. 10.1007/s00038-017-0972-7 28421238 PMC5668328

[19] de Mestral C , Stringhini S. Socioeconomic status and cardiovascular disease. Curr Cardiol Rep 2017;19:115. 10.1007/s11886-017-0917-z 28965316

[20] Raitakari OT , Juonala M, Viikari JS. Obesity in childhood and vascular changes in adulthood: insights into the cardiovascular risk in young finns study. Int J Obes (Lond). 2005;29:S101–4. 10.1038/sj.ijo.0803085 16385760

[21] Norrbäck M , Tynelius P, Ahlström G et al The association of mobility disability and obesity with risk of unemployment in two cohorts from Sweden. BMC Public Health 2019;19:347. 10.1186/s12889-019-6627-2 30922278 PMC6437925

[22] Sweeting HN. Measurement and definitions of obesity in childhood and adolescence: a field guide for the uninitiated. Nutr J 2007;6:32. 10.1186/1475-2891-6-32 17963490 PMC2164947

[23] Jørgensen MB , Thygesen LC, Becker U et al Alcohol consumption and risk of unemployment, sickness absence and disability pension in Denmark: a prospective cohort study. Addiction 2017;112:1754–64. 10.1111/add.13875 28544338

[24] Hallqvist J , Lynch J, Bartley M et al Can we disentangle life course processes of accumulation, critical period and social mobility? An analysis of disadvantaged socio-economic positions and myocardial infarction in the Stockholm heart epidemiology program. Soc Sci Med 2004;58:1555–62. 10.1016/S0277-9536(03)00344-7 14759698

[25] Cerdas S , Härenstam A, Johansson G et al Development of job demands, decision authority and social support in industries with different gender composition—Sweden, 1991-2013. BMC Public Health 2019;19:758. 10.1186/s12889-019-6917-8 31200675 PMC6570932

[26] Statistics Sweden. Women and Men in Sweden—Facts and Figures in Sweden 2022. Örebro: Statics Sweden, 2022.

[27] Nygren K , Gong W, Hammarström A. Is hypertension in adult age related to unemployment at a young age? Results from the Northern swedish cohort. Scand J Public Health 2015;43:52–8. 10.1177/1403494814560845 25431459

[28] Puig-Barrachina V , Giró P, Artazcoz L et al The impact of active labour market policies on health outcomes: a scoping review. Eur J Public Health 2020;30:36–42. 10.1093/eurpub/ckz026 30907412

[29] Sumner RC , Gallagher S. Unemployment as a chronic stressor: a systematic review of cortisol studies. Psychol Health 2017;32:289–311. 10.1080/08870446.2016.1247841 27766906

[30] Russell G , Lightman S. The human stress response. Nat Rev Endocrinol 2019;15:525–34. 10.1038/s41574-019-0228-0 31249398

[31] Lappalainen T , Jurvelin H, Tulppo MP et al Chronotype and metabolic syndrome in midlife: findings from the Northern Finland birth cohort 1966. Am J Physiol Heart Circ Physiol 2024; 327:H38–44. 10.1152/ajpheart.00051.2024 Epub 2024 May 17. 38758129

[32] Henriksen RE , Nilsen RM, Strandberg RB. Loneliness as a risk factor for metabolic syndrome: results from the HUNT study. J Epidemiol Community Health 2019;73:941–6. 10.1136/jech-2019-212335 31266768

[33] Hernán MA , Robins JM. Causal Inference: What If. Boca Raton: Chapman & Hall/CRC, 2020.

