# Supplementary material for: Unemployment during life can lead to metabolic syndrome in adult age. A 40-year follow-up of the Northern Swedish Cohort
Source: Eur J Public Health. 2025 Sep 17;36(2):ckaf166. doi: 10.1093/eurpub/ckaf166 (PMC13017430; doi:10.1093/eurpub/ckaf166)
Supplement: ckaf166_Supplementary_Data [file ckaf166_supplementary_data.docx]

Table S1

The risk of the components of metabolic syndrome in men and women due to unemployment exposure from age 16 to age 56. Logistic regression, adjusted for parents’ socioeconomic status, for obesity and for intake of alcohol at age 16, was used to estimate odds ratios and 95% confidence intervals.

| Component/gender | Odds ratio (95% confidence interval) | |
| --- | --- | --- |
|  | Unadjusted | Adjusted |
| Hypertonia |  |  |
| - men | 1.003 (0.994-1.012) | 1.000 (0.990-1.010) |
| - women | 1.010 (0.999-1.021) | 1.010 (0.999-1.022) |
|  |  |  |
| Central obesity |  |  |
| - men | 1.006 (0.996-1.016) | 1.003 (0.994-1.013) |
| - women | 1.007 (0.994-1.020) | 1.007 (0.994-1.020) |
|  |  |  |
| Hypertriglyceridemia |  |  |
| - men | 1.006 (0.999-1.013) | 1.005 (0.998-1.013) |
| - women | 1.011 (1.001-1.020) | 1.011 (1.001-1.021) |
|  |  |  |
| Low HDL cholesterol |  |  |
| - men | 1.005 (0.998-1.012) | 1.005 (0.998-1.012) |
| - women | 1.016 (1.005-1.026) | 1.016 (1.005-1.026) |
|  |  |  |
| (Pre)diabetes |  |  |
| - men | 1.000 (0.993-1.006) | 1.000 (0.993-1.007) |
| - women | 1.005 (0.996-1.014) | 1.005 (0.996-1.015) |
|  |  | |
